# The interplay between the lysine demethylase KDM1A and DNA methyltransferases in cancer cells is cell cycle dependent

**DOI:** 10.18632/oncotarget.10624

**Published:** 2016-07-16

**Authors:** Carmen Brenner, Judith Luciani, Martin Bizet, Matladi Ndlovu, Eleonore Josseaux, Sarah Dedeurwaerder, Emilie Calonne, Pascale Putmans, Pierre-Francois Cartron, Matthieu Defrance, François Fuks, Rachel Deplus

**Affiliations:** ^1^ Laboratory of Cancer Epigenetics, Faculty of Medicine, ULB-Cancer Research Centre (U-CRC), Université Libre de Bruxelles, 1070 Brussels, Belgium; ^2^ Centre de Recherche en Cancérologie Nantes-Angers, INSERM, U892, Equipe Apoptose et Progression Tumorale, BP7021, 44007 Nantes, France; ^3^ Département de Recherche en Cancérologie, Faculté de Médecine, Université de Nantes, IFR26, F-4400, Nantes, France; ^4^ LaBCT, Institut de Cancérologie de l'Ouest, 44805 Nantes, Saint Herblain Cedex, France

**Keywords:** DNA methylation, histone demethylation, cancer, KDM1A, cell cycle

## Abstract

DNA methylation and histone modifications are key epigenetic regulators of gene expression, and tight connections are known between the two. DNA methyltransferases are upregulated in several tumors and aberrant DNA methylation profiles are a cancer hallmark. On the other hand, histone demethylases are upregulated in cancer cells. Previous work on ES cells has shown that the lysine demethylase KDM1A binds to DNMT1, thereby affecting DNA methylation. In cancer cells, the occurrence of this interaction has not been explored. Here we demonstrate in several tumor cell lines an interaction between KDM1A and both DNMT1 and DNMT3B. Intriguingly and in contrast to what is observed in ES cells, KDM1A depletion in cancer cells was found not to trigger any reduction in the DNMT1 or DNMT3B protein level or any change in DNA methylation. In the S-phase, furthermore, KDM1A and DNMT1 were found, to co-localize within the heterochromatin. Using P-LISA, we revealed substantially increased binding of KDM1A to DNMT1 during the S-phase. Together, our findings propose a mechanistic link between KDM1A and DNA methyltransferases in cancer cells and suggest that the KDM1A/DNMT1 interaction may play a role during replication. Our work also strengthens the idea that DNMTs can exert functions unrelated to act on DNA methylation.

## INTRODUCTION

Current views of human cancer etiology encompass a plethora of events, where both genetic alterations and epigenetic abnormalities play a role. Important epigenetic events include DNA methylation, histone modifications, microRNA synthesis, and nucleosome positioning [1, 2]. Dysregulation of the epigenetic machinery is one of the mechanisms by which many genes preventing abnormal activity, such as DNA repair genes, cell cycle control genes, and apoptosis-promoting genes, are turned off in tumors [2, 3]. This transcriptional silencing is induced notably by aberrant DNA hypermethylation in combination with other chromatin alterations, including decreased activating marks at lysine 4 (H3K4) or lysine 36 (H3K36) of histone H3 and increased repressive marks at lysine 9 (H3K9) or 27 (H3K27) of histone H3 [2, 3].

DNA methyltransferases (DNMTs), which mediate the establishment of DNA methylation patterns and ensure their faithful inheritance in somatic cells, have been identified as crucial players in cancer progression [4–7]. DNMT1 acts primarily as the maintenance DNA methyltransferase during replication, associating with S-phase replication foci [8]. The DNMT3 class enzymes DNMT3A and DNMT3B are mainly viewed as *de novo* DNMTs and are primarily active during embryonic development [9]. Overlapping functions of these enzymes have also been described [4, 10]. Perturbed DNA methylation patterns have been reported in various human cancers, including hepatomas and prostate, colorectal, and breast cancers [11–13]. Elucidating the mechanisms that tightly regulate DNMT functions, stability, and interactions with other proteins is crucial to understanding carcinogenesis.

The N-terminal tails of histones undergo a wide range of modifications, including acetylation, phosphorylation, and methylation. The influence of chromatin structure depends on the type and location of these modifications. In recent years it has become quite clear that DNA methylation and histone modifications are closely interrelated in transcriptional regulation. For example, DNA hypermethylation and histone deacetylation are often associated with silencing of tumor-suppressor genes [14]. The synergistic effects of DNMT and HDAC inhibitors used to reactivate silenced genes lead to clinically measurable responses in patients suffering from acute myeloid leukemia [15, 16] or lung cancer [17].

Close links between DNA methylation and histone methylation have also been evidenced, in the form of interactions between DNMTs and several histone methyltransferases such as Suv39h1/2 and G9a [18, 19]. Through their association with HP1 (Heterochromatin Protein 1), DNMTs are directed to methylated histone H3. DNMTs have also been linked to enzymes capable of removing methyl groups from histones. The first identified histone demethylase, KDM1A, is a lysine-specific demethylase (also known as LSD1, KIAA061, and AOF2) shown to be required for global DNA methylation in ES cells [20]. From histone H3, this enzyme can remove both activating marks (on H3K4) and repressive marks (on H3K9) [21]. KDM1A has been found in various transcription complexes involved in repression, such as CoREST-containing complexes and NuRD [22, 23], or in activation, in complexes where it associates with nuclear androgen or estrogen receptors [24, 25].

A link between KDM1A and DNMTs has been found in embryonic stem cells [20], where KDM1A depletion leads to a gradual decrease in DNA methylation. DNMT1 is known to be methylated by the Set7/9 lysine methyltransferase and demethylated by KDM1A. Set7/9-mediated methylation of DNMT1 leads to its degradation, while direct demethylation by KDM1A increases DNMT1 stability [20].

Many cancer cells are reported to have significantly increased *KDM1A* expression levels [26–28]. In the present study, we have explored for the first time the interplay between KDM1A and DNMTs in cancer cells. We provide evidence that in cancer cells, KDM1A interacts with both DNMT1 and DNMT3B. We find that KDM1A depletion increases the level of dimethylated H3K4 (H3K4Me2) but does not affect the DNA methylation pattern, in contrast to observations on ES cells [20]. We further demonstrate that the KDM1A-DNMT1 interaction is primarily observed during the S-phase, at replication foci. Together, these results demonstrate crosstalk between the lysine demethylase KDM1A and the DNA methyltransferase DNMT1, which could be involved in carcinogenesis independently of its role in DNA methylation.

## RESULTS

### KDM1A interacts with DNMT1 and DNMT3B *in vitro* and also *in vivo* in cancer cells

**Figure 1:**
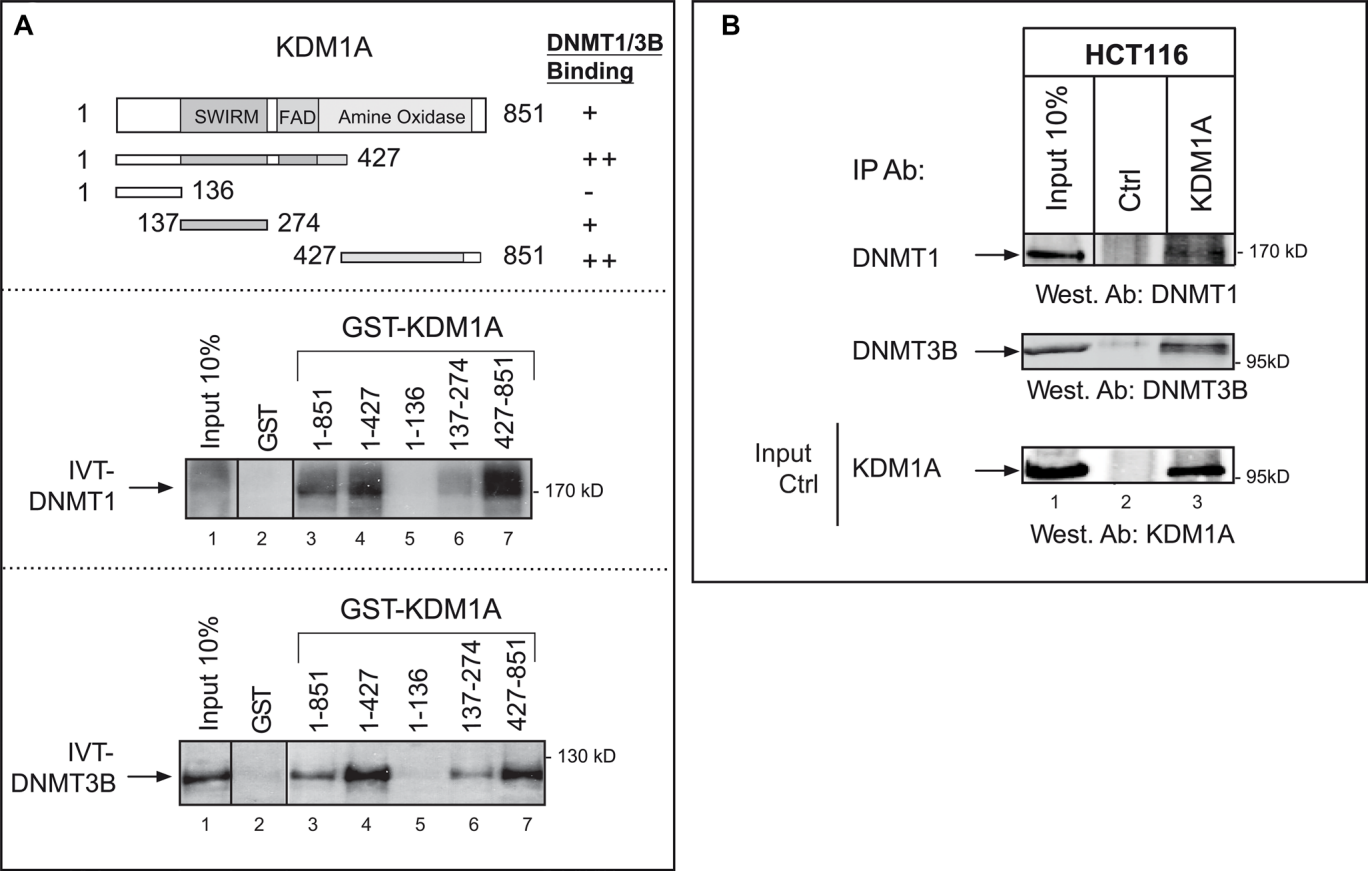
Interaction of KDM1A with DNMT1 and DNMT3B *in vitro* and in cancer cells (**A**) KDM1A interacts with DNMT1 and DNMT3B *in vitro*. Upper panel: Schematic representation of the KDM1A protein, with its known domains highlighted. Also shown are the different sequences that were fused to GST and tested for binding to DNMT1 or DNMT3B. The results are summarized on the right (from “++” [strong interaction] to “–” [no interaction]). Middle and lower panels: The indicated GST fusions were tested in GST pull-down experiments using IVT full-length DNMT1 (IVT-DNMT1) (middle panel) or DNMT3B (IVT-DNMT3B) (lower panel) (lanes 3 to 7). Lane 2 shows the results of the control pull-down with GST protein alone. Lane 1 shows 10% of the radiolabeled IVT-DNMT1 or IVT-DNMT3B engaged in the pull-down experiment. A vertical line indicates juxtaposition of non-adjacent lanes of the same blot (the exposure time was the same). (**B**) Western blots showing that DNMT1 and DNMT3B co-immunoprecipitate with KDM1A from HCT116 whole-cell extracts (lanes 3). Anti-rabbit IgG was used in the negative control (lanes 2). Input stands for non-immunoprecipitated HCT116 extract (10% of the volume subjected to immunoprecipitation) (lanes 1). The western blot at the bottom shows the efficiency of KDM1A immunoprecipitation by the anti-KDM1A antibody. The vertical line indicates juxtaposition of non-adjacent lanes of the same blot (exposure time was the same).

To investigate crosstalk between KDM1A and DNMT in cancer cells, we took advantage of previous observations on mouse ES cells, where DNMT1 has been shown to associate with KDM1A [20]. First, to assess whether KDM1A and DNMT1 associate *in vitro*, we performed a pull-down assay using GST-KDM1A (produced in bacteria) and radiolabeled DNMT1 obtained by *in vitro* translation (Figure 1A, middle panel). In a similar assay, we used DNMT3B instead of DNMT1 (Figure 1A, bottom panel). In these experiments, KDM1A was found to associate with both DNMT1 and DNMT3B. These interactions appeared specific, as none was observed between DNMT1 or DNMT3B and the GST protein alone (Figure 1A, lanes 2) or between KDM1A and an unrelated protein (Supplementary Figure S1). Because KDM1A is known to interact with other proteins through several functional domains, we examined which domain might be involved in DNMT binding. The regions present in the different constructs tested are illustrated in the top panel of Figure 1A. Mapping experiments revealed that the KDM1A SWIRM and amine oxidase domains are required for the interaction with DNMTA or DNMT3B (Figure 1A, lanes 3, 4, 6, and 7). The first 136 N-terminal amino acids of KDM1A, in contrast, proved unnecessary for binding. (Figure 1A, lanes 5). These results are consistent with the solved KDM1A crystal structure, which shows that the N-terminal residues are unstructured and contain nuclear localization signals, while the SWIRM and oxidase domains are intimately bound through an extensive hydrophobic interface [29].

By endogenous co-immunoprecipitation, we found KDM1A to bind DNMT1 and DNMT3B in two different cancer cell lines: when we immunoprecipitated endogenous KDM1A from whole-cell extracts of the colon cancer cell line HCT116, we detected DNMT1 and DNMT3B by western blotting (Figure 1B). The negative control (use of anti-rabbit IgG instead of the anti-KDM1A antibody), only a background signal was observed (Figure 1B: lane 2). Interaction between the KDM1A and DNMT proteins was also shown in HeLa cervical carcinoma cells (Supplementary Figure S2A). Experiments where DNMT1 or DNMT3B was immunoprecipitated and KDM1A revealed by western blotting were also performed. They confirmed that KDM1A associates with both DNMTs (Supplementary Figure S2B)

Previous studies on mouse ES cells have revealed an interaction between KDM1A and DNMT1 only. This suggests that KDM1A may associate with different DNMTs according to the cell context.

### In cancer cells, KDM1A knockdown does not affect levels of DNMT1 or DNMT3B transcripts or of the corresponding proteins

**Figure 2:**
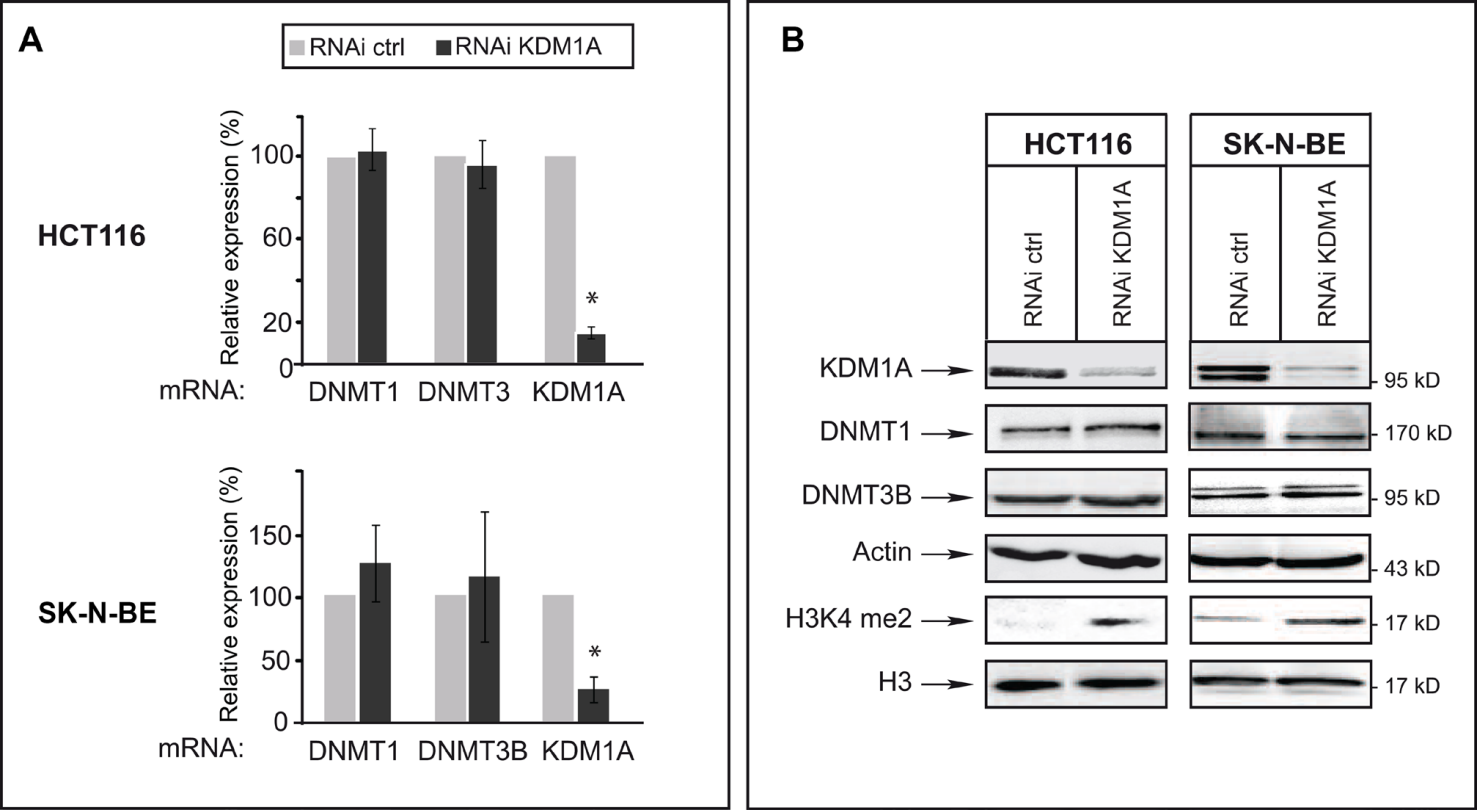
In cancer cells, KDM1A knockdown does not affect levels of DNMT1 or DNMT3B transcripts or of the corresponding proteins (**A**) Quantitative RT-PCR analysis of *KDM1A*, *DNMT1*, and *DNMT3B* expression after RNAi-mediated knockdown of KDM1A in HCT116 and SK-N-BE cells. All transcript levels were normalized to the level observed with the RNAi control. (**B**) KDM1A knockdown in HCT116 and SK-N-BE cells does not affect DNMT protein levels but leads to an increased level of H3K9me2. Western blot analysis performed against KDM1A, DNMTs, and H3K9me2 in both HCT116 and SK-N-BE KDM1A-knockdown cells. Actin and H3 were used as loading controls.

We next examined whether KDM1A knockdown might affect levels of DNMT1 and/or DNMT3B transcripts in cancer cells. For this, we used HCT116 cells and SK-N-BE neuroblastoma cells, as *KDM1A* is known to be highly expressed in neuroblastomas [28, 30]. RT-qPCR revealed an 80% reduced KDM1A mRNA level in KDM1A-knockdown cells, but the levels of DNMT1 and DNMT3B transcripts were unaffected (Figure 2A). We next investigated whether a decrease in KDM1A might affect DNMT protein levels, as observed in ES cells for DNMT1 [20]. Western blots revealed no change in the level of DNMT1 or DNMT3B protein in KDM1A-knockdown cells (Figure 2B and Supplementary Figure S3). Interestingly and in contrast to observations made in ES cells by Wang *et al.* KDM1A knockdown was found to significantly increase the level of H3K4me2 as shown in Figure 2B suggesting that the KDM1A's role is dependant of the cell context. In ES cells the effect of KDM1A on DNMT1 levels is mediated by demethylation of K142 of DNMT1 and affect DNMT1 stability [20, 31]. To explore if DNMT1 is methylated at this lysine residue in cancer cell lines, we realised western blot with an antibody against DNMT1 K142me. As shown in Supplementary Figure S3, DNMT1 is methylated at K142 in cancer cells. Contrary to published data reported in ES cells, DNMT1 methylation and stability is not influenced by the decreased of KDM1A in cancer cells. This results suggest that DNMT1 could be demethylated by another enzyme in cancer cells. In summary, the effect of KDM1A knockdown in the tested cancer cell lines differs from that observed in ES cells in that it does not alter either the levels of these DNMTs, those of the corresponding mRNAs or DNMT methylation.

### In cancer cells, the DNA methylation level is unaffected by KDM1A depletion

We then investigated the impact of KDM1A depletion on the DNA methylation level in cancer cells. In ES cells, KDM1A deficiency causes a global decrease in DNA methylation, in both imprinted genes and major satellite repeats [20]. In normal cells, repetitive sequences usually show a high level of methylation, whereas cancer cells generally exhibit global DNA hypomethylation, primarily in repetitive sequences. This hypomethylation has been linked to genome instability and chromosomal aberrations [3, 32]. To test whether KDM1A-deficient cancer cells show decreased methylation of repetitive sequences, we used pyrosequencing to quantify the methylation of interspersed (LINE1) and tandem repeats (D4Z4, NBL2). DNA methylation analysis of LINE1 repeats, representing ∼20% of the human genome, provides an accurate estimate of global DNA methylation changes [33]. LINE1 hypomethylation has been observed in several types of cancer [34, 35].

**Figure 3:**
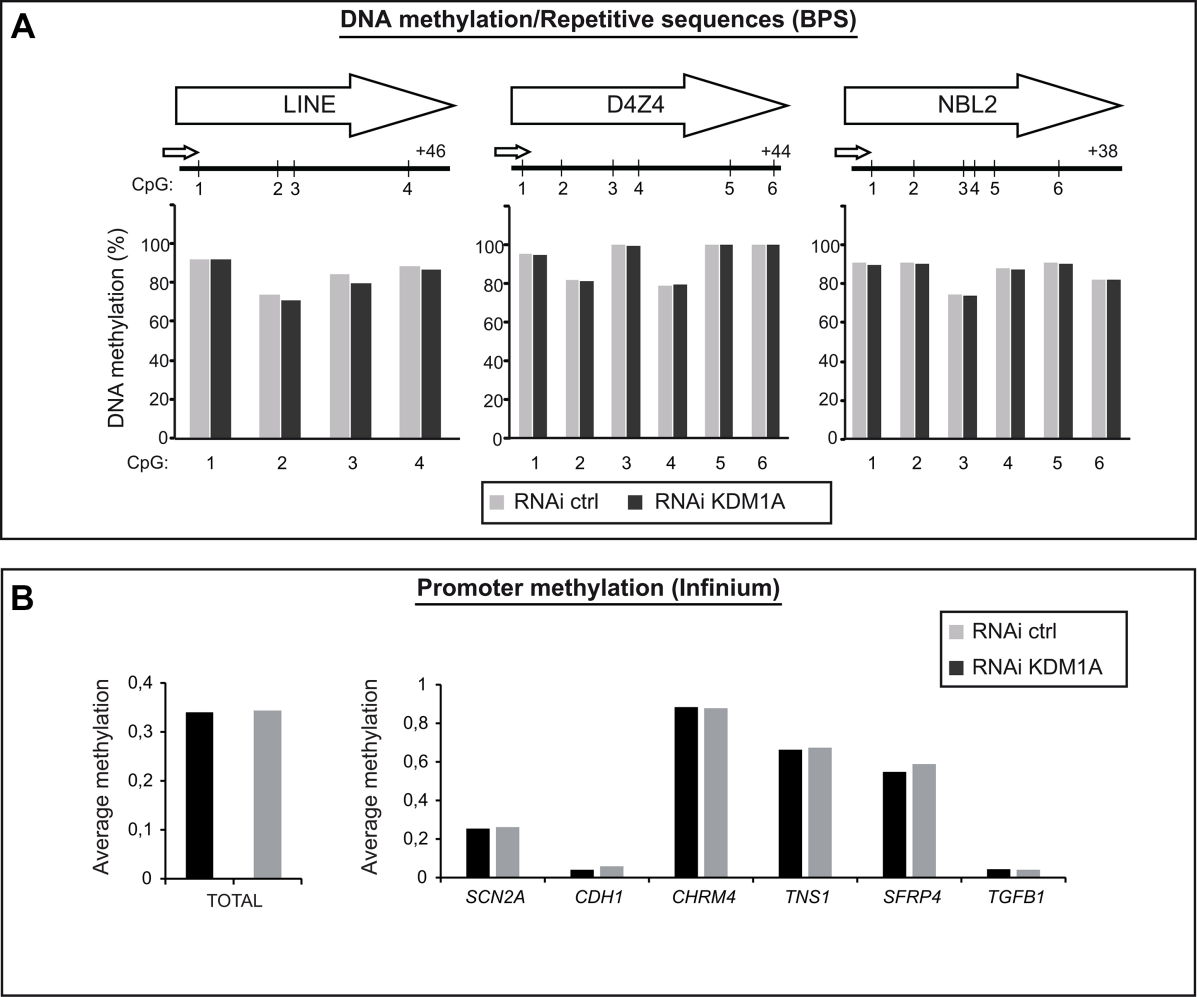
KDM1A does not affect the DNA methylation level in cancer cells (**A**) KDM1A depletion by means of RNA interference is not associated with global DNA demethylation in SK-N-BE cells. Pyrosequencing analysis of interspersed repeats, LINE repeats, and of the tandem repeats NBL2 and D4Z4 shows no difference in DNA methylation level in KDM1A-knockdown versus control cells. (**B**) Promoter methylation analysis performed by means of the Infinium Methylation Assay in SK-NB-E cells treated with RNAi ctrl (grey bar) or RNAi KDM1A (black bar). Left part, histogram comparing the total DNA methylation profiles of SK-N-BE cells treated with a KDM1A-targeting (grey bar) versus a control RNAi (black bar). Right part, methylation average of six known KDM1A target in SK-NB-E cells treated with RNAi ctrl (grey bar) or RNAi KDM1A (black bar).

As shown in Figure 3A, LINE1 repeats appeared highly methylated, as expected, in cells treated with a control RNAi (gray bars) (Figure 3A, left part). KDM1A-depleted cells (RNAi KDM1A, black bars) showed no significant difference in LINE1 DNA methylation as compared to cells treated with the control RNAi. Likewise, KDM1A appeared not to influence the methylation level of the subtelomeric repeats D4Z4 and NBL2 (Figure 3A, middle and right panels), whose methylation is frequently dysregulated in cancer [36].

To analyze DNA methylation more broadly, the Infinium Methylation Assay was used to interrogate the methylation status of 14,475 consensus coding sequences, notably in well-known cancer genes [37]. This analysis again revealed no difference in total promoter DNA methylation between KDM1A-knockown cells and cells treated with the control RNAi (Figure 3B, left panel and Supplementary Figure S4).

We then examined the methylation levels of several known KDM1A target genes: SCN2A, CDH1, CHRM4, TNS1, SFRP4, and TGFB1 [22, 28, 38–40]. Once again, no major methylation change was observed at any of these target sequences (Figure 3B, right panel).

Our results thus indicate that KDM1A acts differently in cancer cells than in ES cells. In the former, in contrast to the latter, this protein does not seem required to maintain DNA methylation levels.

### KDM1A co-localizes with DNMT1 during cell cycle progression

**Figure 4:**
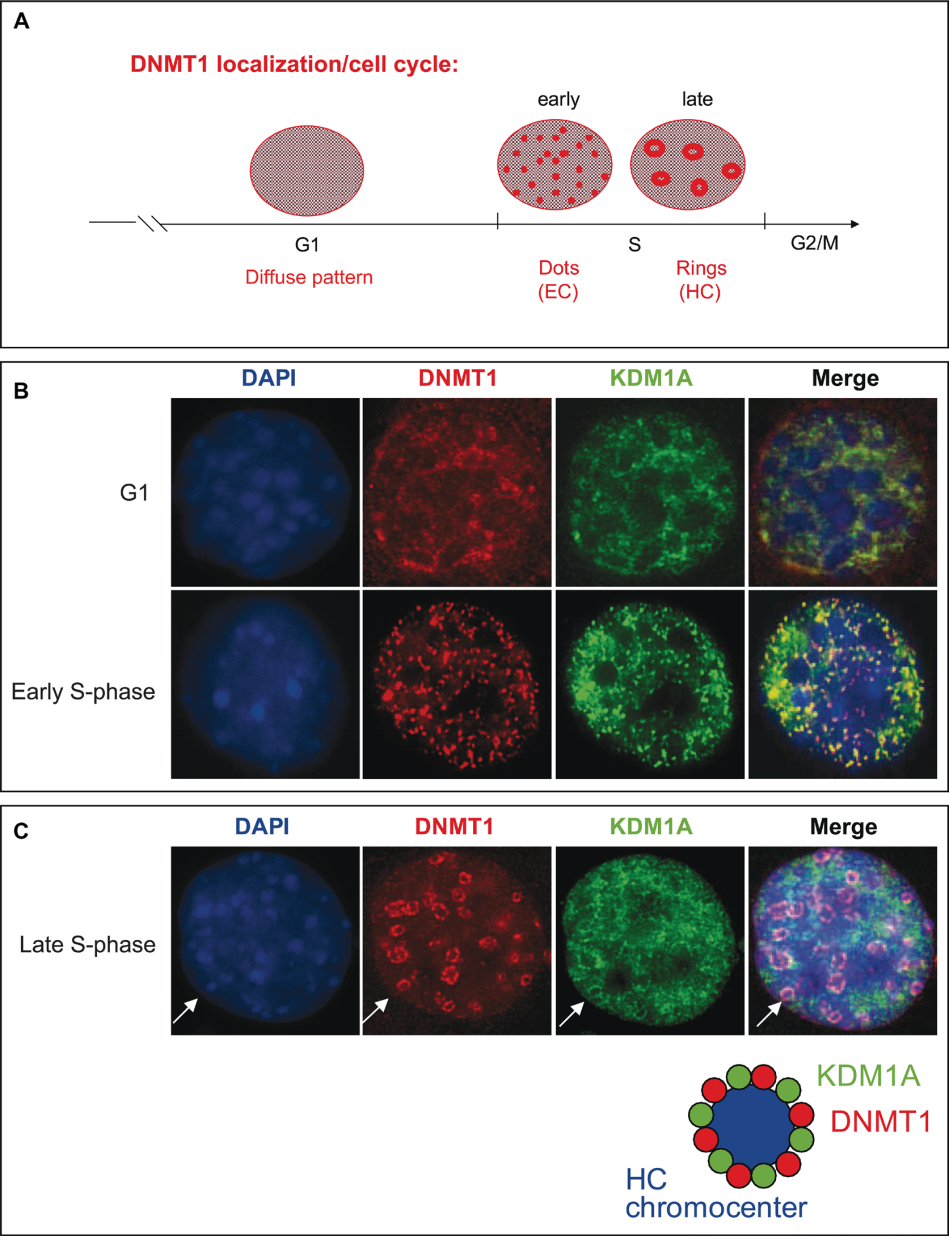
KDM1A co-localizes with DNMT1 during cell cycle progression (**A**) Schematic representation of DNMT1 distribution pattern changes in the course of the cell cycle. In G1 phase a diffuse pattern is observed. In early S-phase, DNMT1 is detected in small, punctate structures (dots), and in late S-phase, ring structures appear [44]. (**B** and **C**) Localization of DNMT1 and KDM1A during the cell cycle. NIH3T3 cells were cotransfected with plasmids expressing myc-DNMT1 and HA-KDM1A. Immunofluorescence staining with antibodies against Myc (red) and KDM1A (green) revealed the respective distributions of these proteins during G1, early S-phase (B), and late S-phase (C). Cell DNA was visualized by DAPI staining.

DNMT1 has recently been reported to form complexes with different partners according to the cell cycle stage [41]. During the S-phase it interacts, mainly at replication forks, with PCNA (Proliferating Cell Nuclear Antigen) and UHRF1 (also known as Np95 and ICBP90), a factor crucial to DNMT1 anchorage to replicating heterochromatin regions. During other cell cycle phases, DNMT1 can target specific genomic regions by binding transcription factors. Schneider *et al.* demonstrate that the dynamics of DNMT1 binding to chromatin is finely regulated during the S-phase. In early S-phase, DNMT1 interacts directly at replication sites, while in late S-phase it interacts predominantly in pericentromeric regions [42]. During cell division, the genome has to be accurately duplicated within the confines of the S-phase, and distinct DNA replication structures are associated with progression through the S-phase of the cell cycle [43]. Notably, the distribution of DNMT1 has been shown to change from diffuse in the G1 phase to a more complex pattern consisting of small, punctate structures in the early S-phase and to ring structures during the middle-to-late S-phase [8, 44, 45] (Figure 4A). While euchromatin regions are transcriptionally active and typically replicated in the early S-phase, when DNMT1 is observed as dispersed dots, heterochromatin regions, where transcription is repressed, are replicated primarily during the late S-phase, when DNMT1 is visible as ring structures (Figure 4A). The significance of DNMT1-containing structures associated with replication sites in each phase of the cell cycle is just emerging. These structures are likely indicative of coupling and cooperation between proteins involved in DNA replication [41, 46].

We first confirmed the identity of the specialized S-phase DNMT1-containing structures observed at replication foci during cell cycle progression. To this end, we used NIH3T3 cells, in which the nuclear localization of DNMTs has been clearly established [45, 47, 48]. To determine the nuclear localization of DNMT1 and PCNA in these cells, we performed immunofluorescence staining for endogenous PCNA on cells transfected with a vector expressing GFP-tagged DNMT1. Consistently with earlier observations [42, 49], DNMT1 and PCNA were found to colocalize at replication foci. The characteristic structural changes observed were considered to assign sub-stages cell cycle phases (Supplementary Figure S5).

We then examined whether KDM1A might associate with specialized DNMT1-containing S-phase structures during replication. HA-KDM1A and Myc-tagged DNMT1 were co-expressed in non-synchronized NIH3T3 cells to determine their nuclear distribution. As shown in Figure 4B and 4C, two populations of co-transfected cells were observed: cells in G1 phase, as indicated by a diffuse nucleoplasmic distribution of both DNMT1 (red), with KDM1A (green) showing a similar distribution, and cells progressing through the S-phase, as demonstrated by the characteristic nuclear DNMT1 distribution patterns. At S-phase onset, Myc-DNMT1 was found to localize to small punctate structures broadly distributed throughout the nuclei (Figure 4B), while as the cells progressed further through the S-phase, Myc-DNMT1 showed a distinct partial or large ring structure at DAPI-dense dots indicative of late S-phase, when heterochromatin replication occurs (Figure 4C). Strikingly, HA-KDM1A exhibited the same sequence of distribution patterns associated with progression through the S-phase. When the two staining patterns were merged, a partial overlap between KDM1A and DNMT1 was observed (orange) at structures called heterochromatin chromocenters (Figure 4C). This evidence suggesting that KDM1A co-localizes with DNMT1 at sites of active DNA synthesis in S-phase nuclei prompted us to further investigate whether KDM1A and DNMT1 interact directly, and at which phases of the cell cycle. The DNMT1-KDM1A colocalisation is partial as also observed in the Supplementary Figure S6A. Probably, only a significant part, of the cellular pool of KDM1A and DNMT1 are involved in this interaction. A portion of cellular DNMT1 binds KDM1A and the remaining is part of other complex as for instance with SP1. These two partners have then common but also independent implications.

### In cancer cells, directly interacting DNMT1 and KDM1A accumulate in the S-phase

**Figure 5:**
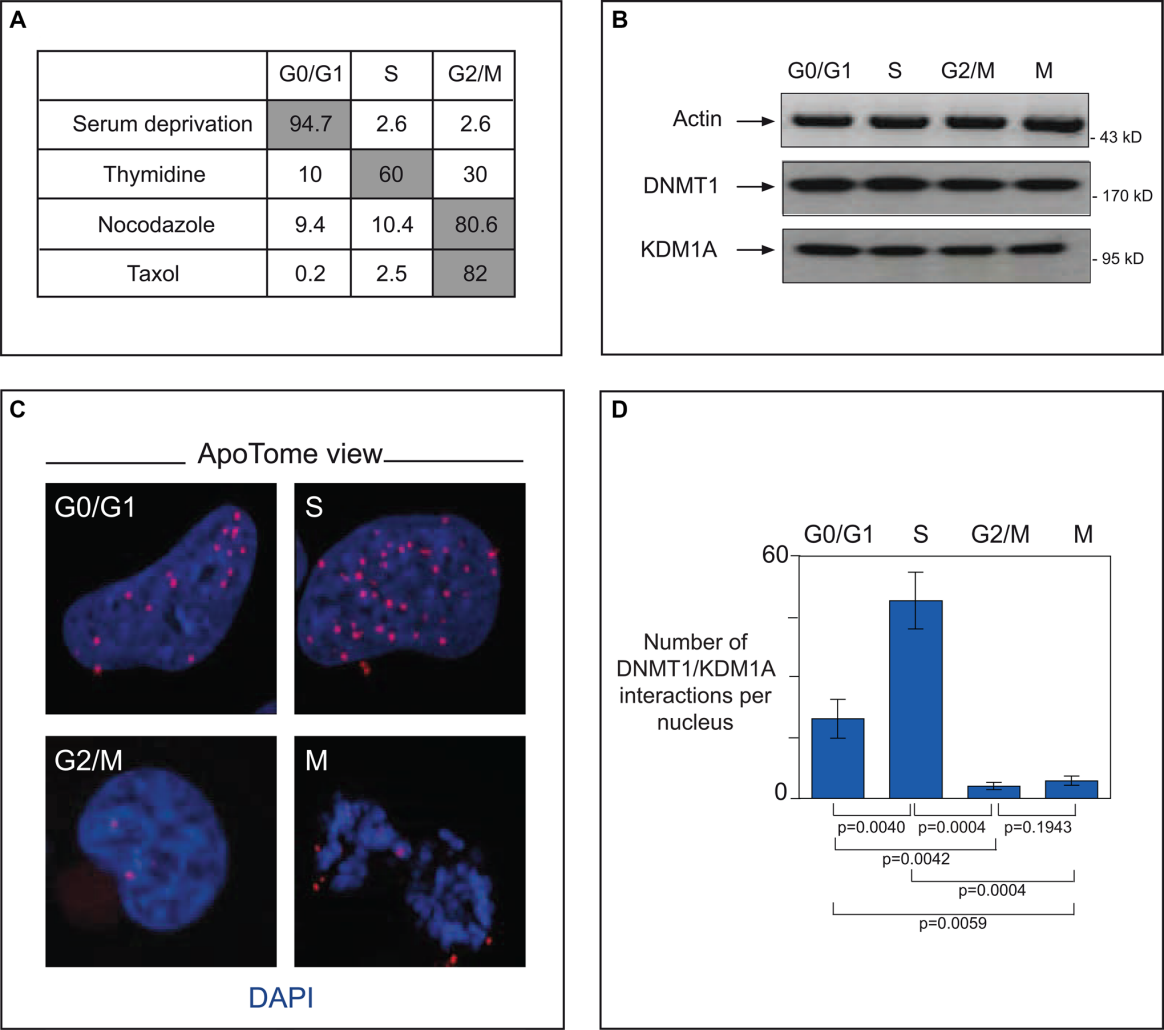
In cancer cells, interacting DNMT1 and KDM1A accumulate in the S-phase (**A**) Cell cycle phase analysis of synchronized glioma U251 cells. To obtain cells blocked in GO/G1 these cells were respectively serum-deprived for 74 h. Enrichment in S-phase-blocked cells was done by exposure to thymidine (2 mM, 24 h). Treatment with nocodazole (100 ng/mL, 24 h), or taxol (150 ng/mL, 24 h) yielded cells blocked in G2/M. Cell cycle analyses were done with a NucleoCounter NC-3000. Results of a representative experiment are shown (**B**) Endogenous protein levels in synchronized U251 cells. Western blot detection of actin (MAB1501R, Milipore), DNMT1 (sc10221,Tebu-Bio), and KDM1A (sc67672, Tebu-Bio) after cell synchonization. (**C**) ApoTome view of DNMT1-KDM1A interactions in U251 cells during the different phases, as visualized by P-LISA (Olink, Bioscience). DNMT1/KDM1A interactions, detected with antibodies against DNMT1 (sc10221, Tebu-Bio) and KDM1A (sc67672, Tebu-Bio), appear as red dots. Nuclear DNA is stained in blue with DAPI (ProLong^®^ Gold antifade reagent with DAPI, Invitrogen). (**D**) Graph (means ± SD) illustrating the number of DNMT1-KDM1A interactions per nucleus in U251 cells during the different phases of the cell cycle. The number of DNMT1/PCNA interactions is calculated from the analysis of at least 50 nuclei in three independent experiments.

KDM1A has been implicated in several cancer types, particularly neural tumors [28, 30, 50]. Having observed that DNMT1 and KDM1A co-localize at replication foci in NIH3T3 cells, we wondered whether they interact directly in cancer cells. To answer this question, we used serum starvation, thymidine treatment, or nocodazole (or taxol) treatment to synchronize U251 glioma cells in G1, S, or G2/M, respectively. As expected, cell cycle phase analysis showed most (up to 94.7%) of the serum-deprived cells to be blocked in GO/G1 and a majority of thymidine-treated cells (60%) to be blocked in S-phase. Nocodazole treatment stopped up to 80% of the treated cells in phase G2/M, while taxol treatment caused 82% of the cells to be blocked in mitosis (Figure 5A). Endogenous levels of the DNMT1 and KDM1A proteins remained constant throughout the cell cycle, as indicated by western blot analysis (Figure 5B).

Using these synchronized cells as tools, we then tackled the question of whether DNMT1 and KDM1A interact directly during cell division. For this we used the Proximity Ligation *In Situ* Assay (P-LISA) and ApoTome technology (Figure 5C and 5D). P-LISA is a technique allowing highly specific and sensitive direct identification of protein interactions [41]. In brief, two primary antibodies from different species recognize the two proteins suspected of interacting. Two species-specific secondary antibodies, each with a unique short DNA strand (PLA probe) attached to it, bind to the primary antibodies. When the PLA probes are in close proximity, they can interact. After amplification by a polymerase, amplicons are detected by means of fluorochrome-coupled DNA probes (Supplementary Figure S7). Visualization with ApoTome technology increases the resolution of captured pictures. This approach enabled us to demonstrate direct interaction between DNMT1 and KDM1A in the nucleus (red dots) during the S-phase (Figure 5C). Overlap with standard DNMT1 immunofluorescence confirm that P-LISA spots are where DNMT1 is localised. Significantly fewer spots indicative of this interaction were observed in cell phases other than S: only about half as many in G0/G1 (*p* = 0.004) and about a tenth as many in G2/M and M (*p* = 0.004 and *p* = 0,004 respectively) (Figure 5D). Specificity of the KDM1A-DNMT1 interaction was checked by experiments performed with only one antibody (Supplementary Figure S6B) and P-LISA experiments in cells in which DNMT1 is down-regulated by RNAi (Supplementary Figure S6C). Taken together, our results indicate that KDM1A associates specifically and directly with DNMT1 in a cell-cycle-dependent manner.

## DISCUSSION

In the context of chromatin, DNA methylation does not function in isolation. It has become clear that there is a complex interplay between DNA methylation and histone modifications, including acetylation, methylation, and ubiquitination of N-terminal histone tails. In particular, lysine methylation machineries are interconnected and rely mechanistically on DNA methylation to promote normal chromatin function [51]. For example, DNMTs interact with several histone methyltransferases, such as G9a and Suv39h1/2 [18, 52, 53]. Furthermore, DNMTs are recruited to H3K9-methylated heterochromatin through direct interaction with HP1 (Heterochromatin Protein 1), which binds H3K9Me3, and the enzymatic activity of Suv39h1/2 is required to establish DNA methylation in pericentric heterochromatin [19, 52]. DNA methyltransferases also interact with KDM1A, as demonstrated in ES cells [20]. KDM1A-deprived ES cells display significantly reduced DNA methylation [20], and KDM1A increases DNMT1 stability [20]. Several connexions between DNA methylation and histone modifications have been described in cancer, but the link between KDM1A and DNMTs in cancer cells has not been explored.

Aberrant DNA methylation is a well-characterized epigenetic hallmark of several cancers [1, 54–56]. Dysregulated expression of *DNMT* genes has been reported for various human tumors [57]. Oncogenic properties of KDM1A have been described in numerous cancers, including prostate cancer [58], lung cancer [59], to neuroblastoma [28]. Furthermore, KDM1A inhibition reduces tumor growth, while its overexpression can contribute to human carcinogenesis [27]. In this work, we present evidence that KDM1A interacts with DNMTs in cancer cells.

In mouse ES cells, KDM1A is required for DNA methylation [20]. We show here that in cancer cells, this is not the case. Furthermore, we have observed no decrease in DNMT1 protein in KDM1A-knockdown cancer cells, suggesting that KDM1A might not affect the stability of the DNMT1 protein in the context of cancer. Our results show that KDM1A knockdown in cancer cells increases global H3K4 dimethylation, while in KDM1A-knockout ES cells, no change in H3K4 dimethylation was seen. Additionally, we have observed no changes in DNA methylation in repetitive sequences or gene promoters in KDM1A-knockdown versus control cells (Figure 3 and Supplementary Figure S4). The observed epigenetic differences between cancer cells and ES cells might not be so surprising. ES and cancer cells are distinct cell types, even though they share common features [60, 61]. Notably, their DNA methylation signatures are different. The regulation of imprinted genes by epigenetic mechanisms is also different in ES and cancer cells [62]. In addition, H3K4 methylation, an activation mark, is strongly diminished in adult cancer cells, whereas ES cells show both H3K4 and H3K27 methylation [61]. The modalities of the interplay between KDM1A and DNMTs thus probably depend on cell features.

Our data fit with the notion that DNMTs act as recruitment platforms for other regulatory machineries, and that some of their functions do not require their catalytic activity [63–65]. DNMTs could mediate transcriptional repression in a DNA-methylation-independent way in stress responses [66] or cancer [67]. In line with this view, an inhibitor of DNMT, 5-aza-2′-deoxycitidine, has been shown to increase expression of target genes without affecting their methylation [68].

The connection between DNMT1 and KDM1A could be of clinical value, for instance in cancer therapy. DNMT and KDM1A inhibitors are well characterized and already used in clinical research [69–71]. Treatments combining DNMT inhibitors with other drugs acting on epigenetic machineries, such as histone deacetylase inhibitors, have already shown therapeutic advantages [15]. In the future, it should be interesting to test whether combining DNMT inhibitors with KDM1A inhibitors can be beneficial to patients in cancer treatment.

Our immunofluorescence results imply that active histone demethylation is closely associated with DNMTs during the establishment and maintenance of epigenetic states. We have demonstrated co-localization of KDM1A with DNMT1 in all the established patterns typical of the S-phase, highlighting the presence of both proteins at replication sites from early to late S-phase, as replication occurs first in euchromatin and then in heterochromatin. Remarkably, our P-LISA experiments demonstrate direct interaction between DNMT1 and KDM1A (Figure 5C), particularly during the S-phase, when chromatin remodeling, DNA methylation, centromeric heterochromatin formation, and chromatin assembly occur [72].

The fact that both DNMT1 and KDM1A have been found, independently, to promote cell cycle progression, including through a replication barrier [73, 74], suggests that crosstalk between histone demethylation and DNA methylation could be essential in enabling cancer cells to overcome cell cycle checkpoints. In S-phase, the DNMT1/PCNA/UHRF1 complex is recruited to DNA [41]. Fine analysis of DNMT1 binding to DNA has revealed specific interactions at replication sites in early S-phase, followed by stronger binding to pericentromeric heterochromatin in late S-phase [42]. In a fission yeast, KDM1A has been shown to control replication fork pauses and imprinting [73]. There is emerging evidence of active loss of histone lysine 4 methylation (H3K4me) during heterochromatin formation. Studies on *Saccharomyces cerevisiae* [72] have shown that under specific stimuli, removal of the stable chromatin mark H3K4me3 through active histone demethylation occurs during the S-phase. Furthermore, this rapid erasure is dynamically regulated at early- and late-replicating genomic loci during heterochromatin assembly. Deletion of another histone demethylase, Jhd2/KDM5, results in dramatically reduced H3K4me3 erasure at most loci [75]. It is therefore reasonable to suggest that KDM1A might act as an extra regulatory layer in the control of chromatin formation and replication. DNMT1, known to recruit the corepressor DMAP1 [44], could be necessary to recruit KDM1A to replication foci in the S-phase. This would mean that H3K4 demethylase activity participates in the global epigenetic modification process taking place at this stage of the cell cycle. Future functional studies are needed to shed more light on the exact role of the KDM1A-DNMT1 interaction during cell cycle progression and on its impact on cancer development. How catalytic activities of KDM1A and DNMT1 and their interaction are regulated during cell cycle? Other actors are involved? Our work could lead to a better understanding of epigenetic cell cycle control in carcinogenesis.

## MATERIALS AND METHODS

### GST fusion, *in vitro* translation, and pull-down assays

Recombinant proteins were produced in and purified from *E. coli* as described [76]. The TNT system (Promega) was used to carry out *in vitro* transcription/translation. DNMT1 and DNMT3B were *in vitro* translated, respectively, from the pcDNA3-GAL4-DNMT1 or pcDNA3-GAL4-DNMT3B plasmid. Recombinant proteins were expressed in and purified from *E. coli* BL21 as described [76]. We used the TNT system (Promega) to carry out *in vitro* transcription/translation. GST pull-down experiments were performed as previously described [76]. Briefly, equivalent amount of GST proteins are incubated with ^35^S-IVTproteins in 200 μl of Z' buffer (25 mM HEPES pH 7.5, 300 mM KCl, 12.5 mM MgCl_2_, 20% glycerol, 0.1% Igepal) added of 1 μL DTT 200 mM and 6 μl BSA (5 mg/ml) during one hour at room temperature. Complexes are washed five times with 1 ml of NETN buffer (20 mM Tris pH8, 100 mM NaCl, 1 mM EDTA, 0.5% Igepal) and loaded on a polyacrylamide gel as well as 10% of the ^35^S-IVT proteins (input).

### Cell culture, drug treatment, and RNAi-mediated knockdown

HeLa, SK-N-BE, and 293 GP cells were maintained in DMEM (Sigma) supplemented with 10% fetal calf serum (Invitrogen). HCT116 cells were grown in McCoy medium supplemented with 10% fetal calf serum. U251 cells were cultured in EMEM with added glutamine (2 mM), non-essential amino acids (1%), sodium pyruvate (1 mM), and fetal calf serum (10%). For cell synchronization, cells were subjected to different treatments: serum deprivation for 72 h to obtain cells in the G0/G1 phase, thymidine treatment (2 mM for 24 h) to increase the number of cells in the S-phase, or treatment for 24 h with Nocodazole (100 ng/ml) or Taxol (150 ng/ml) to block cells in G2/M. Cell cycle phase analyses were performed with a cell counter. RNAi KDM1A plasmid construct production, retroviral particle production in 293T GP cells, and infections were carried out as previously described [77]. Briefly, 293 GP cells were transfected with pRS retroviral vectors and supernatants were collected and used to infect target cells. ES cells were kindly provided by Dr. Li, Dr. Chen and Dr. Hardikar and were maintained as previously described [20].

### Immunoprecipations and Western blot analyses

Whole-cell extracts were prepared using 300 μl IPH lysis buffer (50 mM Tris-HCl pH 8, 150 mM NaCl, 5 mM EDTA, 0.5% NP40 added of antiproteases tablettes (Roche) during 1 hour and centrifuged at 10 000 g for 5 min. All procedures were performed at 4°C. Standard procedures were used for immunoprecipitations and western blotting. Briefly, 2 μG of antibody were added to cellular extract and incubated at 4°C on wheel overnight. Then, 40 μl of A and G sepharose beads were added and samples were incubated for two additional hours. Samples were the washed five time with IPH buffer and loaded on SDS polyacrylamide gel.

Anti-KDM1A (ab17721,Abcam), anti-DNMT1 (ab19905, Abcam), anti- DNMT1 K142me (kindly provided by Shirahsa Pradhan), anti-DNMT3B (ab2851, Abcam), anti-actin (A5316, Sigma), anti-H3K4me2 (07–030, Millipore), anti-H3 (ab1791, Abcam), and anti-rabbit IgG (sc2027; Santa Cruz), were used in immunoprecipitation assays and western blot analyses.

### RNA purification and RT-PCR analysis

Extraction of mRNA was done with the Qiagen RNeasy Mini Kit according to the manufacturer's instructions. DNase treatment was performed with a DNA-free DNase kit (Ambion) according to the manufacturer's instructions. mRNA was reverse transcribed and PCR was performed on the obtained cDNA as described previously [76]. Results are normalized with respect to endogenous control and are the mean of three independent experiments. Primer sequences are available upon request.

### DNA extraction and bisulfite pyrosequencing

Genomic DNA was extracted with the QIAamp DNA Mini Kit (Qiagen). The recommended proteinase K and RNase A digestions were included. Genomic DNA (1 μg) was then bisulfite-converted with the EpiTect Bisulfite Kit (Qiagen). Approximately half of the converted DNA was used as template in each subsequent PCR. Primers for PCR amplification and sequencing were designed with the PyroMark^®^ Assay Design 2.0 software (Qiagen). PCRs were performed with the HotStarTaq DNA polymerase PCR kit (Qiagen) under the following conditions: 95°C 15 min; 55 cycles of [95°C 30 s; 50°C 1 min; 72°C 1 min]; 72°C 10 min. The amplification level was assessed by agarose gel electrophoresis and pyrosequencing of the PCR products was performed with the Pyromark™ Q24 system (Qiagen). All primer sequences are available upon request.

### Illumina Infinium^®^ methylation assay

Genomic DNA was extracted as described above. Site-specific CpG methylation was analyzed by means of the Infinium^®^ HumanMethylation27 bead-array-based technique. This array was developed to assay 27,578 CpG sites selected from over 14,000 genes. The Zymo EZ DNA Methylation KitTM (Zymo Research, Orange, USA) was used to treat 1 μg genomic DNA with sodium bisulfite. This was done according to the manufacturer's procedure, with the alternative incubation conditions recommended when using the Illumina Infinium^®^ Methylation Assay. The assay was performed on 4 μL converted gDNA at 50 ng/μL concentration, according to the protocol described in the Infinium^®^ Methylation Assay Manual. Raw Infinium data were filtered by removing low quality data using a detection *P*-value threshold of 0.05. As the vast majority of Illumina HumanMethylation27 targets are covered by the HumanMethylation450 we used the extended annotation provided by Price *et al.* [78] to filter out the Probes containing SNPs (see [11] for a detailed description). Probes associated to X and Y chromosomes were removed from the analysis. β-values were computed using the formula β-value = M/[U + M] where M and U are the raw “methylated” and “unmethylated” signals, respectively.

### Immunofluorescence analysis

NIH3T3 embryonic fibroblasts were grown on coverslips in DMEM supplemented with 10% FBS. The cells were transfected with the indicated expression plasmid. Transfections were performed in OptiMem medium and Fugene transfection reagent (Roche) according to the manufacturer's recommendations. Cells were incubated for 5 h post-transfection, after which the OptiMem medium was replaced with fresh DMEM. 24 h post-transfection, the cells were washed in PBS and fixed in 1% paraformaldehyde for 10 min at RT. After one wash in PBS, the cells were permeabilized twice for 5 min in PBS containing 0.5% Triton X-100. A blocking step was then performed for 1 h at RT with PBS containing 1% BSA.

Incubation with the different antibodies diluted in blocking solution was performed for 1.5 h at 37°C. We used mouse anti-Myc (Santa Cruz, sc-40) at 1/200 dilution, rabbit anti-KDM1A (2139, Cell Signaling) at 1/50 dilution, and mouse anti-PCNA (M0879, DAKO) at 1/300 dilution. When cells were observed, the major difference in KDM1A staining intensity allowed unambiguous discrimination of cells transfected with the KDM1A expression construct from those expressing only endogenous KDM1A. After three PBS washes, secondary antibodies diluted in blocking solution were added and incubated for 1 h at RT: we used Cy3-tagged anti-mouse antibody (CLCC35010, Cederlane) at 1:4000 dilution and FITC-tagged anti-rabbit antibody (ab6717, Abcam) at 1/1000 dilution. Coverslips were washed three times with PBS, incubated in DAPI solution (500 ng/μL) for 3 min, and washed again. The cells were then mounted on glass microscope slides in Vectashield mounting medium (Vector). To detect PCNA, the same protocol was used, except for the initial fixation step, in which the cells were fixed in cold methanol for 10 min at –20°C and the coverslips incubated in acetone for 1 min at RT. The permeabilization and blocking steps were as described above. The specificity of each experiment was tested by omitting the primary antibody or by using non-transfected cells as negative controls.

### Proximity ligation *in situ* assay (P-LISA)

Cells were cultured on coverslips for 24 h and then fixed with 4% paraformaldehyde in PBS pH 7.4 for 15 min at room temperature. Permeabilization was performed for 20 min at RT with 0.5% Triton 100× in PBS. The blocking, staining, hybridization, ligation, amplification, and detection steps were carried out according to the manufacturer's instructions (Olink Bioscience). During these steps, all incubations were performed in a humidity chamber. We used anti-KDM1A (sc-67272) and anti-DNMT1 (sc10221) for P-LISA and anti DNMT1 (sc 271729) for strandars immunofluorescence presented in Supplementary Figure S6A). The amplification and detection steps were performed in a dark room. Fluorescence was visualized with the Axiovert 200M microscopy system (Zeiss, LePecq, France) equipped with the ApoTome module (X63 and numerical aperture 1.4). Preparations were mounted in ProLong Gold antifade reagent with DAPI (InVitrogen, France). Picture acquisition was done by structured illumination microscopy. The lateral resolution (rl), according to the Rayleigh criterion, was: rl = 0.61λ/NA (λ: wavelength; NA: numerical aperture of the objective), while the axial resolution, ApoTome, was defined by the full-width half maximum (FWHMz)

FWHM(z)=3.8316π⋅λ×10−3n⋅sin2(α2)ν¯(1−ν¯2)

In this equation, λ is the emission wavelength, n is the index of medium refraction, ν the frequency, and α the opening angle of the objective as previously described [79]. After 9 deconvolvings with the software Huygens Essential 3.5 (SVI), 3D images were obtained with the Amira.4.1.1 program. Finally, the images were analyzed with the freeware BlobFinder, available for download from www.cb.uu.se/∼amin/BlobFinder. In other words, the use of this program allows normalization, standardization and reproducibility and also makes it possible to define the cut-off signal for accepting/quantifying a dot or not.

## SUPPLEMENTARY MATERIALS FIGURES



## References

[1] Robertson KD (2005). DNA methylation and human disease. Nat Rev Genet.

[2] Ting AH, McGarvey KM, Baylin SB (2006). The cancer epigenome—components and functional correlates. Genes Dev.

[3] Jones PA, Baylin SB (2007). The epigenomics of cancer. Cell.

[4] Rhee I, Bachman KE, Park BH, Jair KW, Yen RWC, Schuebel KE, Cui H, Feinberg AP, Lengauer C, Kinzler KW, Baylin SB, Vogelstein B (2002). DNMT1 and DNMT3b cooperate to silence genes in human cancer cells. Nature.

[5] Mutze K, Langer R, Schumacher F, Becker K, Ott K, Novotny A, Hapfelmeier A, Höfler H, Keller G (2011). DNA methyltransferase 1 as a predictive biomarker and potential therapeutic target for chemotherapy in gastric cancer. Eur J Cancer.

[6] Rajendran G, Shanmuganandam K, Bendre A, Muzumdar D, Mujumdar D, Goel A, Shiras A (2011). Epigenetic regulation of DNA methyltransferases: DNMT1 and DNMT3B in gliomas. J Neurooncol.

[7] Brenner C, Fuks F (2006). DNA methyltransferases: facts, clues, mysteries. Curr Top Microbiol Immunol.

[8] Leonhardt H, Page AW, Weier HU, Bestor TH (1992). A targeting sequence directs DNA methyltransferase to sites of DNA replication in mammalian nuclei. Cell.

[9] Okano M, Bell DW, Haber DA, Li E (1999). DNA methyltransferases Dnmt3a and Dnmt3b are essential for de novo methylation and mammalian development. Cell.

[10] Fatemi M, Hermann A, Gowher H, Jeltsch A (2002). Dnmt3a and Dnmt1 functionally cooperate during de novo methylation of DNA. Eur J Biochem.

[11] Dedeurwaerder S, Desmedt C, Calonne E, Singhal SK, Haibe-Kains B, Defrance M, Michiels S, Volkmar M, Deplus R, Luciani J, Lallemand F, Larsimont D, Toussaint J (2011). DNA methylation profiling reveals a predominant immune component in breast cancers. EMBO Mol Med.

[12] Revill K, Wang T, Lachenmayer A, Kojima K, Harrington A, Li J, Hoshida Y, Llovet JM, Powers S (2013). Genome-wide methylation analysis and epigenetic unmasking identify tumor suppressor genes in hepatocellular carcinoma. Gastroenterology.

[13] Lao VV, Grady WM (2011). Epigenetics and colorectal cancer. Nat Rev Gastroenterol Hepatol.

[14] Baylin SB (2005). DNA methylation and gene silencing in cancer. Nat Clin Pract Oncol.

[15] Gore SD (2005). Combination therapy with DNA methyltransferase inhibitors in hematologic malignancies. Nat Clin Pr Oncol.

[16] Griffiths EA, Gore SD (2008). DNA methyltransferase and histone deacetylase inhibitors in the treatment of myelodysplastic syndromes. Semin Hematol.

[17] Vendetti FP, Rudin CM (2013). Epigenetic therapy in non-small-cell lung cancer: targeting DNA methyltransferases and histone deacetylases. Expert Opin Biol Ther.

[18] Epsztejn-Litman S, Feldman N, Abu-Remaileh M, Shufaro Y, Gerson A, Ueda J, Deplus R, Fuks F, Shinkai Y, Cedar H BY (2008). De novo DNA methylation promoted by G9a prevents reprogramming of embryonically silenced genes. Nat Struct Mol Biol.

[19] Fuks F, Hurd PJ, Deplus R, Kouzarides T (2003). The DNA methyltransferases associate with HP1 and the SUV39H1 histone methyltransferase. Nucleic Acids Res.

[20] Wang J, Hevi S, Kurash JK, Lei H, Gay F, Bajko J, Su H, Sun W, Chang H, Xu G, Gaudet F, Li E, Chen T (2009). The lysine demethylase LSD1 (KDM1) is required for maintenance of global DNA methylation. Nat Genet.

[21] Nicholson TB, Chen T (2009). LSD1 demethylates histone and non-histone proteins. Epigenetics.

[22] Wang Y, Zhang H, Chen Y, Sun Y, Yang F, Yu W, Liang J, Sun L, Yang X, Shi L, Li R, Li Y, Zhang Y (2009). LSD1 is a subunit of the NuRD complex and targets the metastasis programs in breast cancer. Cell.

[23] Shi YJ, Matson C, Lan F, Iwase S, Baba T, Shi Y (2005). Regulation of LSD1 Histone Demethylase Activity by Its Associated Factors. Mol Cell.

[24] Pollock JA, Larrea MD, Jasper JS, McDonnell DP, McCafferty DG (2012). Lysine-specific histone demethylase 1 inhibitors control breast cancer proliferation in ERα-dependent and -independent manners. ACS Chem Biol.

[25] Cai C, He HH, Chen S, Coleman I, Wang H, Fang Z, Chen S, Nelson PS, Liu XS, Brown M, Balk SP (2011). Androgen receptor gene expression in prostate cancer is directly suppressed by the androgen receptor through recruitment of lysine-specific demethylase 1. Cancer Cell.

[26] Cho HS, Suzuki T, Dohmae N, Hayami S, Unoki M, Yoshimatsu M, Toyokawa G, Takawa M, Chen T, Kurash JK, Field HI, Ponder BAJ, Nakamura Y (2011). Demethylation of RB regulator MYPT1 by histone demethylase LSD1 promotes cell cycle progression in cancer cells. Cancer Res.

[27] Hayami S, Kelly JD, Cho HS, Yoshimatsu M, Unoki M, Tsunoda T, Field HI, Neal DE, Yamaue H, Ponder BAJ, Nakamura Y, Hamamoto R (2011). Overexpression of LSD1 contributes to human carcinogenesis through chromatin regulation in various cancers. Int J Cancer.

[28] Schulte JH, Lim S, Schramm A, Friedrichs N, Koster J, Versteeg R, Ora I, Pajtler K, Klein-Hitpass L, Kuhfittig-Kulle S, Metzger E, Schule R, Eggert A (2009). Lysine-specific demethylase 1 is strongly expressed in poorly differentiated neuroblastoma: implications for therapy. Cancer Res.

[29] Tochio N, Umehara T, Koshiba S, Inoue M, Yabuki T, Aoki M, Seki E, Watanabe S, Tomo Y, Hanada M, Ikari M, Sato M, Terada T (2006). Solution structure of the SWIRM domain of human histone demethylase LSD1. Structure.

[30] Amente S, Milazzo G, Sorrentino MC, Ambrosio S, Di Palo G, Lania L, Perini G, Majello B (2015). Lysine-specific demethylase (LSD1/KDM1A) and MYCN cooperatively repress tumor suppressor genes in neuroblastoma. Oncotarget.

[31] Estève PO, Chang Y, Samaranayake M, Upadhyay AK, Horton JR, Feehery GR, Cheng X, Pradhan S (2011). A methylation and phosphorylation switch between an adjacent lysine and serine determines human DNMT1 stability. Nat Struct Mol Biol.

[32] Tost J (2010). DNA methylation: an introduction to the biology and the disease-associated changes of a promising biomarker. Mol Biotechnol.

[33] Yang AS, Estécio MRH, Doshi K, Kondo Y, Tajara EH, Issa JPJ (2004). A simple method for estimating global DNA methylation using bisulfite PCR of repetitive DNA elements. Nucleic Acids Res.

[34] Hsiung DT, Marsit CJ, Houseman EA, Eddy K, Furniss CS, McClean MD, Kelsey KT (2007). Global DNA methylation level in whole blood as a biomarker in head and neck squamous cell carcinoma. Cancer Epidemiol Biomarkers Prev.

[35] Wilhelm CS, Kelsey KT, Butler R, Plaza S, Gagne L, Zens MS, Andrew AS, Morris S, Nelson HH, Schned AR, Karagas MR, Marsit CJ (2010). Implications of LINE1 methylation for bladder cancer risk in women. Clin Cancer Res.

[36] Choi SH, Worswick S, Byun HM, Shear T, Soussa JC, Wolff EM, Douer D, Garcia-Manero G, Liang G, Yang AS (2009). Changes in DNA methylation of tandem DNA repeats are different from interspersed repeats in cancer. Int J Cancer.

[37] Bibikova M, Le J, Barnes B, Saedinia-Melnyk S, Zhou L, Shen R, Gunderson KL (2009). Genome-wide DNA methylation profiling using Infinium^®^ assay. Epigenomics.

[38] Shi Y, Lan F, Matson C, Mulligan P, Whetstine JR, Cole PA, Casero RA, Shi Y (2004). Histone demethylation mediated by the nuclear amine oxidase homolog LSD1. Cell.

[39] Ding D, Liu X, Guo SW (2014). Overexpression of lysine-specific demethylase 1 in ovarian endometriomas and its inhibition reduces cellular proliferation, cell cycle progression, and invasiveness. Fertil Steril.

[40] Wang J, Lu F, Ren Q, Sun H, Xu Z, Lan R, Liu Y, Ward D, Quan J, Ye T, Zhang H (2011). Novel Histone Demethylase LSD1 Inhibitors Selectively Target Cancer Cells with Pluripotent Stem Cell Properties. Cancer Res.

[41] Hervouet E, Nadaradjane A, Gueguen M, Vallette FM, Cartron PF (2012). Kinetics of DNA methylation inheritance by the Dnmt1-including complexes during the cell cycle. Cell Div.

[42] Schneider K, Fuchs C, Dobay A, Rottach A, Qin W, Wolf P, Álvarez-Castro JM, Nalaskowski MM, Kremmer E, Schmid V, Leonhardt H, Schermelleh L (2013). Dissection of cell cycle-dependent dynamics of Dnmt1 by FRAP and diffusion-coupled modeling. Nucleic Acids Res.

[43] O'Keefe RT, Henderson SC, Spector DL (1992). Dynamic organization of DNA replication in mammalian cell nuclei: spatially and temporally defined replication of chromosome-specific alpha-satellite DNA sequences. J Cell Biol.

[44] Rountree MR, Bachman KE, Baylin SB (2000). DNMT1 binds HDAC2 and a new co-repressor, DMAP1, to form a complex at replication foci. Nat Genet.

[45] Bachman KE, Rountree MR, Baylin SB (2001). Dnmt3a and Dnmt3b are transcriptional repressors that exhibit unique localization properties to heterochromatin. J Biol Chem.

[46] Probst A V, Dunleavy E, Almouzni G (2009). Epigenetic inheritance during the cell cycle. Nat Rev Mol Cell Biol.

[47] Zhang J, Gao Q, Li P, Liu X, Jia Y, Wu W, Li J, Dong S, Koseki H, Wong J (2011). S phase-dependent interaction with DNMT1 dictates the role of UHRF1 but not UHRF2 in DNA methylation maintenance. Cell Res.

[48] Deplus R, Blanchon L, Rajavelu A, Boukaba A, Defrance M, Luciani J, Rothé F, Dedeurwaerder S, Denis H, Brinkman AB, Simmer F, Müller F, Bertin B (2014). Regulation of DNA methylation patterns by CK2-mediated phosphorylation of Dnmt3a. Cell Rep.

[49] Schönenberger F, Deutzmann A, Ferrando-May E, Merhof D (2015). Discrimination of cell cycle phases in PCNA-immunolabeled cells. BMC Bioinformatics.

[50] Singh MM, Manton CA, Bhat KP, Tsai WW, Aldape K, Barton MC, Chandra J (2011). Inhibition of LSD1 sensitizes glioblastoma cells to histone deacetylase inhibitors. Neuro Oncol.

[51] Hashimoto H, Vertino PM, Cheng X (2010). Molecular coupling of DNA methylation and histone methylation. Epigenomics.

[52] Lehnertz B, Ueda Y, Derijck AAHA, Braunschweig U, Perez-Burgos L, Kubicek S, Chen T, Li E, Jenuwein T, Peters AHFM (2003). Suv39h-mediated histone H3 lysine 9 methylation directs DNA methylation to major satellite repeats at pericentric heterochromatin. Curr Biol.

[53] Smallwood A, Estève PO, Pradhan S, Carey M (2007). Functional cooperation between HP1 and DNMT1 mediates gene silencing. Genes Dev.

[54] Keil KP, Vezina CM (2015). DNA methylation as a dynamic regulator of development and disease processes: spotlight on the prostate. Epigenomics.

[55] Dedeurwaerder S, Fuks F (2012). DNA methylation markers for breast cancer prognosis: Unmasking the immune component. Oncoimmunology.

[56] Lee EJ, Rath P, Liu J, Ryu D, Pei L, Noonepalle SK, Shull AY, Feng Q, Litofsky NS, Miller DC, Anthony DC, Kirk MD, Laterra J (2015). Identification of Global DNA Methylation Signatures in Glioblastoma-Derived Cancer Stem Cells. J Genet Genomics.

[57] Miremadi A, Oestergaard MZ, Pharoah PDP, Caldas C (2007). Cancer genetics of epigenetic genes. Hum Mol Genet.

[58] Kahl P, Gullotti L, Heukamp LC, Wolf S, Friedrichs N, Vorreuther R, Solleder G, Bastian PJ, Ellinger J, Metzger E, Schüle R, Buettner R (2006). Androgen receptor coactivators lysine-specific histone demethylase 1 and four and a half LIM domain protein 2 predict risk of prostate cancer recurrence. Cancer Res.

[59] Lv T, Yuan D, Miao X, Lv Y, Zhan P, Shen X, Song Y (2012). Over-expression of LSD1 promotes proliferation, migration and invasion in non-small cell lung cancer. PLoS One.

[60] Altun G, Loring JF, Laurent LC (2010). DNA methylation in embryonic stem cells. J Cell Biochem.

[61] Ohm JE, McGarvey KM, Yu X, Cheng L, Schuebel KE, Cope L, Mohammad HP, Chen W, Daniel VC, Yu W, Berman DM, Jenuwein T, Pruitt K (2007). A stem cell-like chromatin pattern may predispose tumor suppressor genes to DNA hypermethylation and heritable silencing. Nat Genet.

[62] Bibikova M, Chudin E, Wu B, Zhou L, Garcia EW, Liu Y, Shin S, Plaia TW, Auerbach JM, Arking DE, Gonzalez R, Crook J, Davidson B (2006). Human embryonic stem cells have a unique epigenetic signature. Genome Res.

[63] Fuks F, Burgers WA, Godin N, Kasai M, Kouzarides T (2001). Dnmt3a binds deacetylases and is recruited by a sequence-specific repressor to silence transcription. EMBO J.

[64] Fuks F (2005). DNA methylation and histone modifications: teaming up to silence genes. Curr Opin Genet Dev.

[65] Burgers WA, Fuks F, Kouzarides T (2002). DNA methyltransferases get connected to chromatin. Trends Genet.

[66] Milutinovic S, Brown SE, Zhuang Q, Szyf M (2004). DNA methyltransferase 1 knock down induces gene expression by a mechanism independent of DNA methylation and histone deacetylation. J Biol Chem.

[67] Haney SL, Hlady RA, Opavska J, Klinkebiel D, Pirruccello SJ, Dutta S, Datta K, Simpson MA, Wu L, Opavsky R (2015). Methylation-independent repression of Dnmt3b contributes to oncogenic activity of Dnmt3a in mouse MYC-induced T-cell lymphomagenesis. Oncogene.

[68] Huang Y, Stewart TM, Wu Y, Baylin SB, Marton LJ, Perkins B, Jones RJ, Woster PM, Casero RA (2009). Novel oligoamine analogues inhibit lysine-specific demethylase 1 and induce reexpression of epigenetically silenced genes. Clin Cancer Res.

[69] Schenk T, Chen WC, Göllner S, Howell L, Jin L, Hebestreit K, Klein HU, Popescu AC, Burnett A, Mills K, Casero RA, Marton L, Woster P (2012). Inhibition of the LSD1 (KDM1A) demethylase reactivates the all-trans-retinoic acid differentiation pathway in acute myeloid leukemia. Nat Med.

[70] Harris WJ, Huang X, Lynch JT, Spencer GJ, Hitchin JR, Li Y, Ciceri F, Blaser JG, Greystoke BF, Jordan AM, Miller CJ, Ogilvie DJ, Somervaille TCP (2012). The histone demethylase KDM1A sustains the oncogenic potential of MLL-AF9 leukemia stem cells. Cancer Cell.

[71] Fahy J, Jeltsch A, Arimondo PB (2012). DNA methyltransferase inhibitors in cancer: a chemical and therapeutic patent overview and selected clinical studies. Expert Opin Ther Pat.

[72] Katan-Khaykovich Y, Struhl K (2005). Heterochromatin formation involves changes in histone modifications over multiple cell generations. EMBO J.

[73] Holmes A, Roseaulin L, Schurra C, Waxin H, Lambert S, Zaratiegui M, Martienssen RA, Arcangioli B (2012). Lsd1 and lsd2 control programmed replication fork pauses and imprinting in fission yeast. Cell Rep.

[74] Chen T, Hevi S, Gay F, Tsujimoto N, He T, Zhang B, Ueda Y, Li E (2007). Complete inactivation of DNMT1 leads to mitotic catastrophe in human cancer cells. Nat Genet.

[75] Seward DJ, Cubberley G, Kim S, Schonewald M, Zhang L, Tripet B, Bentley DL (2007). Demethylation of trimethylated histone H3 Lys4 *in vivo* by JARID1 JmjC proteins. Nat Struct Mol Biol.

[76] Fuks F, Burgers WA, Brehm A, Hughes-Davies L, Kouzarides T (2000). DNA methyltransferase Dnmt1 associates with histone deacetylase activity. Nat Genet.

[77] Denis H, Deplus R, Putmans P, Yamada M, Métivier RFF (2009). Functional connection between deimination and deacetylation of histones. Mol Cell Biol.

[78] Price ME, Cotton AM, Lam LL, Farré P, Emberly E, Brown CJ, Robinson WP, Kobor MS (2013). Additional annotation enhances potential for biologically-relevant analysis of the Illumina Infinium HumanMethylation450 BeadChip array. Epigenetics Chromatin.

[79] Hervouet E, Hulin P, Vallette FM, Cartron P-F (2011). Proximity ligation *in situ* assay for monitoring the global DNA methylation in cells. BMC Biotechnol.

