# Supplementary material for: The interplay between the lysine demethylase KDM1A and DNA methyltransferases in cancer cells is cell cycle dependent
Source: Oncotarget. 2016 Jul 16;7(37):58939–52. doi: 10.18632/oncotarget.10624 (PMC5312287; doi:10.18632/oncotarget.10624)
Supplement: Supplementary file 1 [file oncotarget-07-58939-s001.pdf]

The interplay between the lysine demethylase KDM1A and DNA methyltransferases in cancer cells is cell cycle dependent

Supplementary Materials

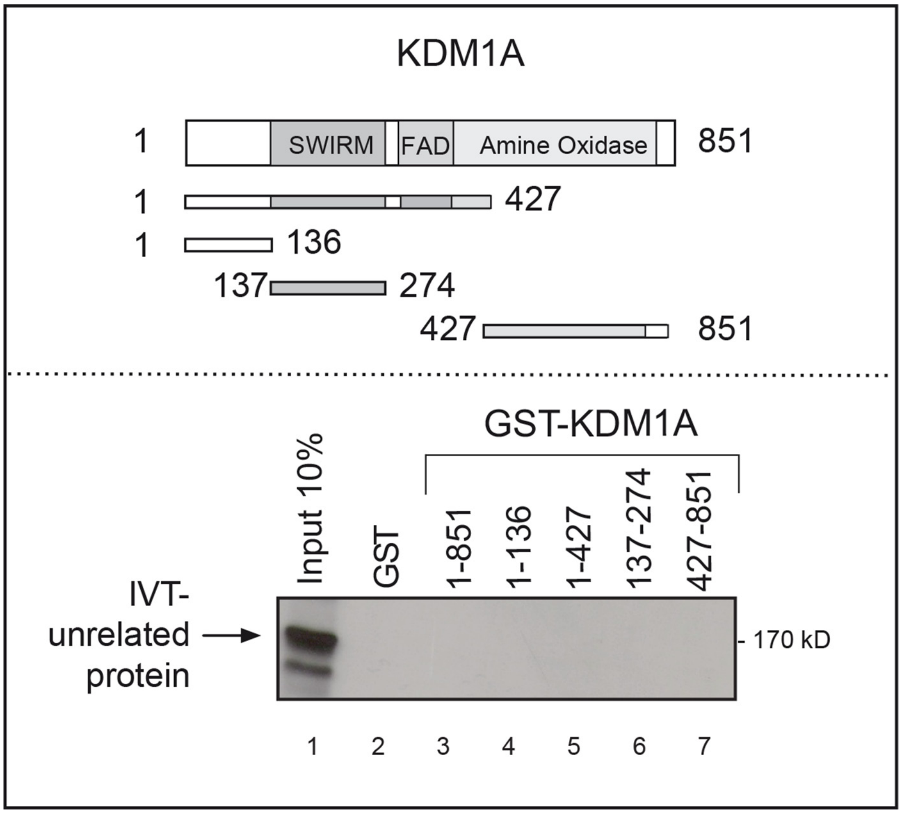

Supplementary Figure S1: (related to Figure 1): KDM1A doesn't interact with an unrelated protein *in vitro*. Upper panel: Schematic representation of the KDM1A protein, with its known domains highlighted. Lower panels: The indicated GST fusions were tested in GST pull-down experiments using IVT full-length unrelated protein (IVT-unrelated protein). Lane 2 shows the results of the control pull-down with GST protein alone. Lane 1 shows 10% of the radiolabeled IVT-unrelated protein engaged in the pull-down experiment.

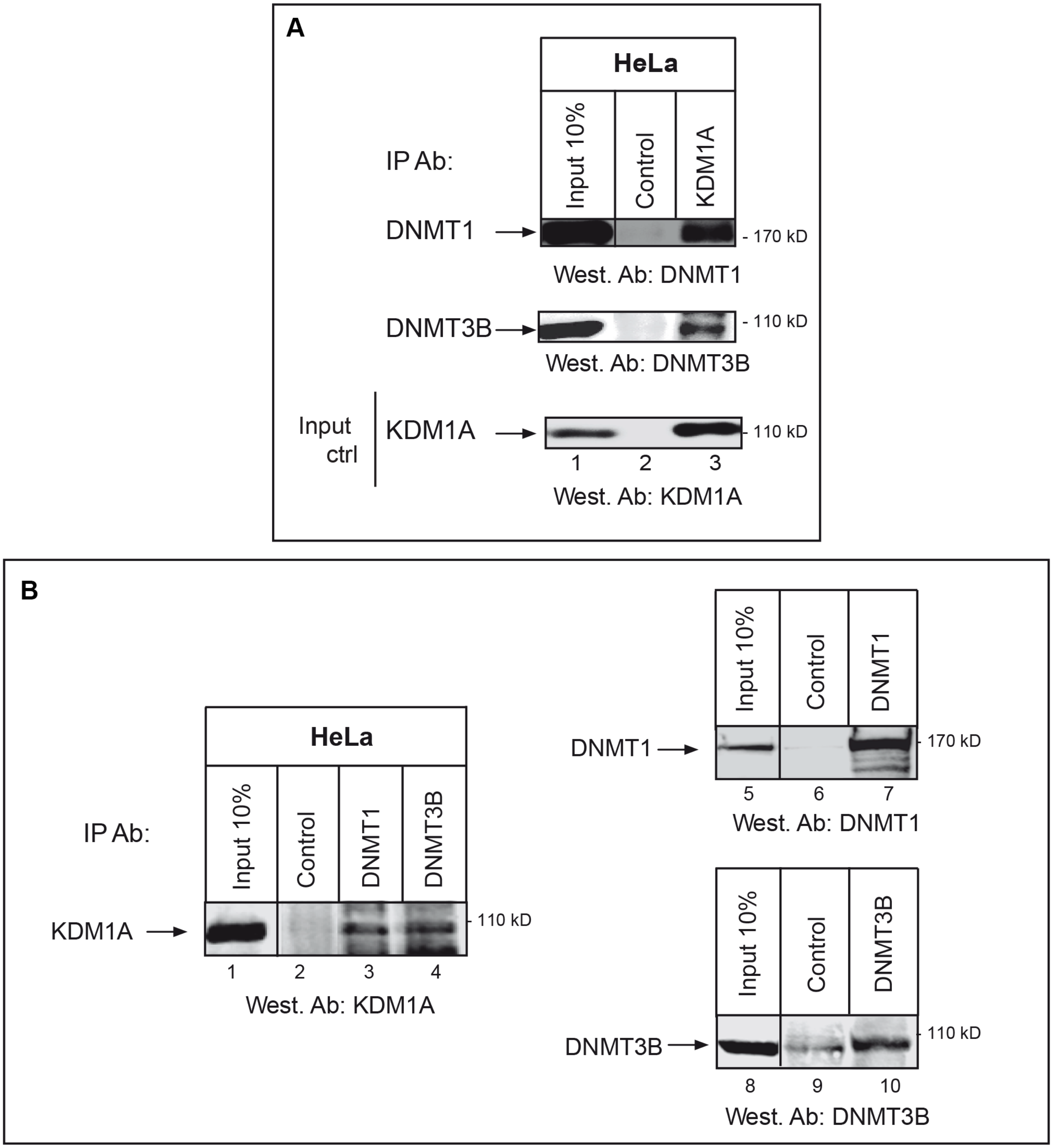

Supplementary Figure S2: (related to Figure 1): KDM1A interacts with DNMT1 and DNMT3B in HeLa cells. (**A**) DNMT1 and DNMT3B co-immunoprecipitate with KDM1A from HeLa nuclear extracts. In the control, anti-rabbit IgG was used instead of anti-KDM1A (lane 2). Input stands for the non-immunoprecipitated HeLa nuclear extract (10% of the volume used in the immunoprecipitation). The efficiency of KDM1A immunoprecipitation is visualized by western blotting with anti- KDM1A antibody (Input ctrl). (**B**) KDM1A coimmunoprecipitates with DNMT1 and DNMT3B from HeLa nuclear extracts (lanes 3 and 4). Anti-rabbit IgG was used in the control (lane 2). Input stands for the non-immunoprecipitated HeLa nuclear extract (10% of the volume used in the immunoprecipitation) (lane 1). Right part: The efficiency of immunoprecipitaion is visualized on the western blots probed with anti-DNMT1 (lane 7) and anti-DNMT3B (lane 10). The vertical line indicates juxtaposition of non-adjacent lanes of the same blot (exposure time was the same).

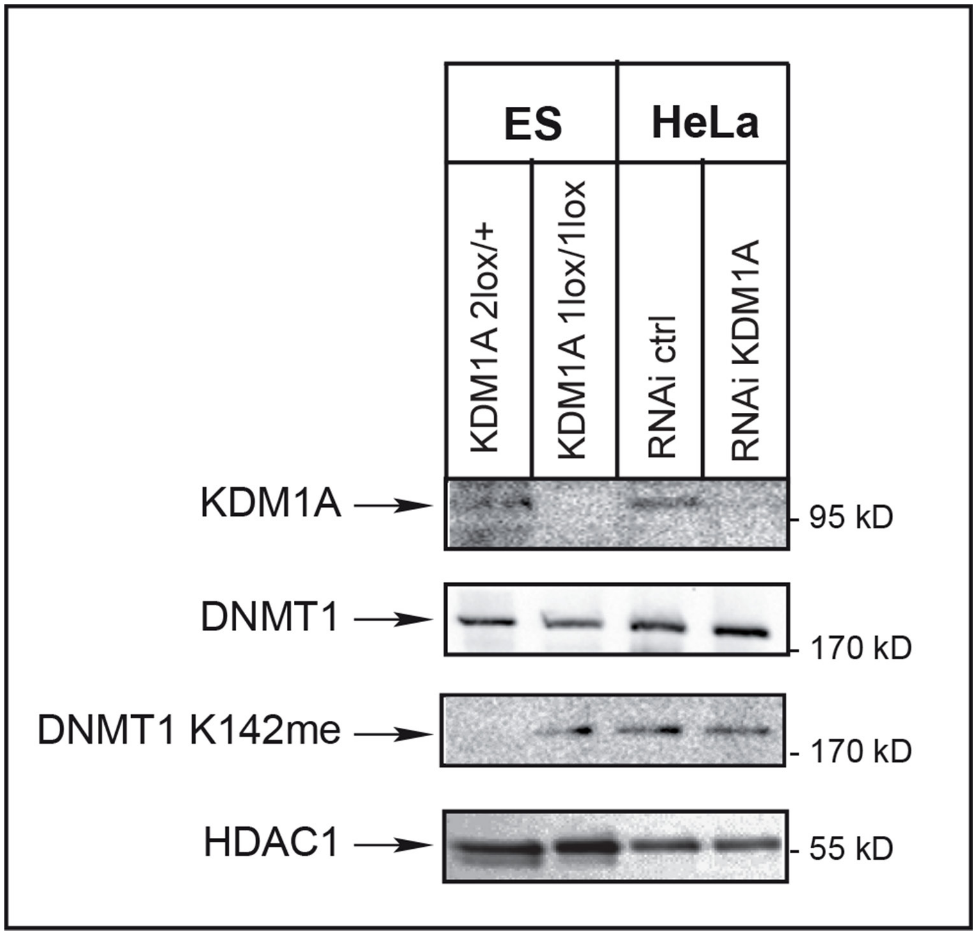

Supplementary Figure S3: (related to Figure 2): Contrary to ES cells, KDM1A downreulation in HeLa cells does not affect DNMT1 protein levels or DNMT1 methylation. Western blot analysis performed against KDM1A, DNMT1, DNMT1 K142me and HDAC1 in both HeLa KDM1A-knockdown cells and ES KDM1A knockout cells. HDAC1 was used as loading controls.

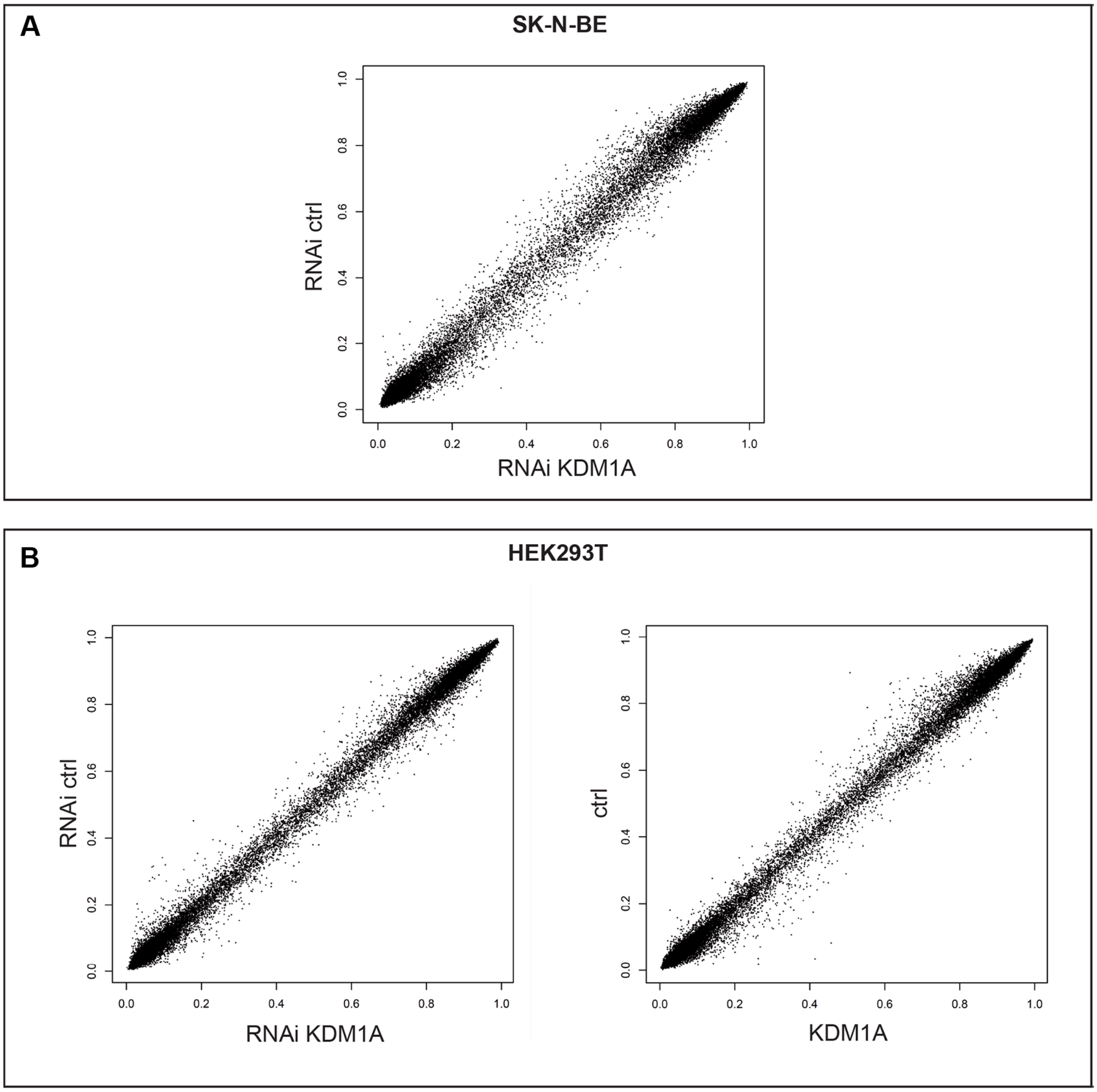

Supplementary Figure S4: (related to Figure 2D): modulation of KDM1A does not induces change in DNA methylation in cancer cell lines. (**A**)Scatter-plot analyses comparing the DNA methylation profiles in SK-NB-E cells treated with RNAi ctrl or RNAi KDM1A. (**B**) Left panel, Scatter-plot analyses comparing the DNA methylation profiles in HEK293T cells treated with RNAi ctrl or RNAI KDM1A.or right panel, in HEK293T overexpressing empty vector or KDM1A. DNA methylation analysis were performed as previously described (see Material and Methods)

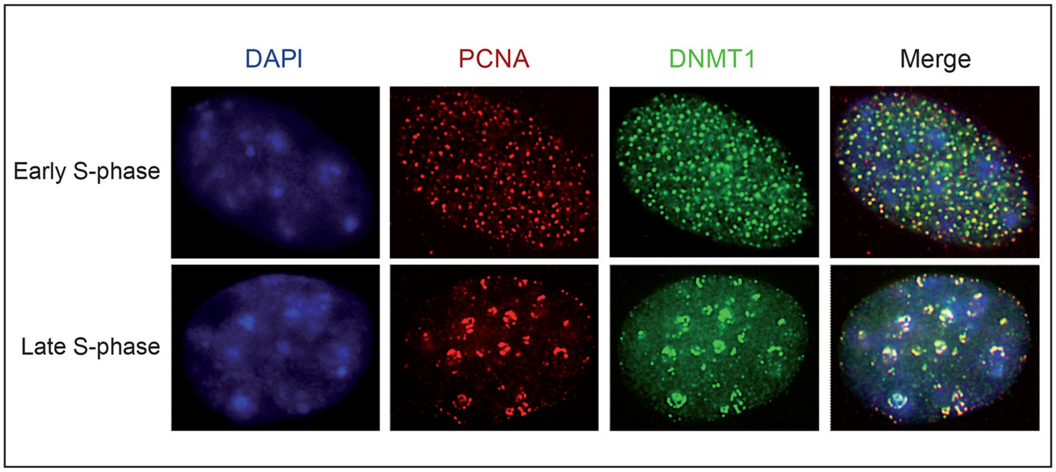

Supplementary Figure S5: DNMT1 co-localizes with PCNA at replication foci throughout the S-phase. NIH3T3 cells were transfected with GFP-DNMT1, stained with anti-PCNA (red), and observed by fluorescence microscopy to determine the distributions of DNMT1 and PCNA during early S-phase (upper part) and late Sphase (lower part). Cell DNA was visualized by DAPI staining (blue).

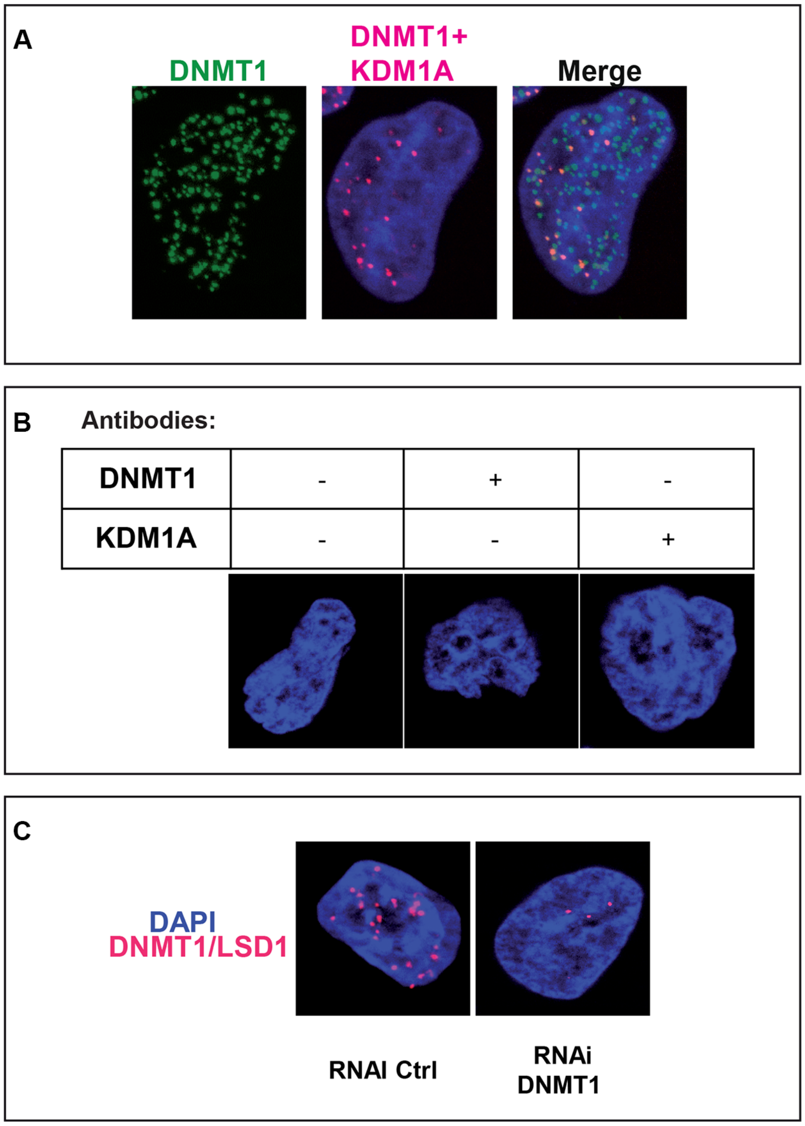

Supplementary Figure S6: (related to Figure 5): Control of KDM1A-DNMT1 interaction. (**A**) P-LISA KDM1A-DNMT1 spots are overlaping with conventionnal DNMT1 imunofluorence imaging. (**B**) No P-LISA signal was detected when experimentations were performed with only one antibody. (**C**) P-LISA signal relative to DNMT1-LSD1 is strongly reduced in presence of RNAi DNMT1.

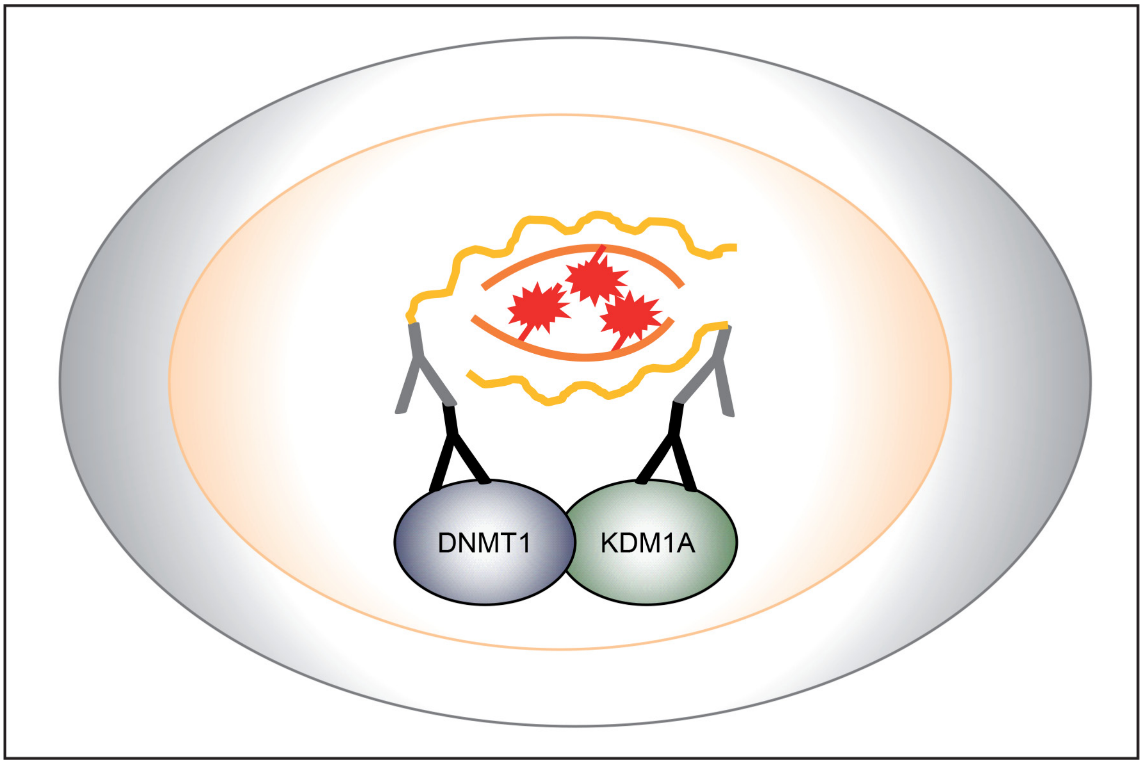

Supplementary Figure S7: Schematic representation of proximity ligation *in situ* assay (P-LISA). In this technique, specific primary antibodies from different species are used to detect direct interaction between two proteins. Species-specific secondary antibodies are coupled with a short single strand nucleic sequence. If proteins of interest interact, two probes are rather close, allowing their binding and amplification by a polymerase is possible. Amplicon detection is done with DNA probes coupled to a fluorochrome.
